# An assessment of the impact of formal preparation activities on performance in the University Clinical Aptitude Test (UCAT): a national study

**DOI:** 10.1186/s12909-022-03811-y

**Published:** 2022-10-28

**Authors:** Sanat Kulkarni, Jayne Parry, Alice Sitch

**Affiliations:** Institute of Applied Health Research, University of Birmingham, Edgbaston, Birmingham, B15 2TT UK

**Keywords:** UCAT, Preparation, Coaching, Widening access, Selection

## Abstract

**Background:**

Previous studies have shown performance in the University Clinical Aptitude Test (UCAT) to be associated with measures of candidate socio-economic advantage such as parental occupation and type of school attended. It is possible that access to preparation support and materials may in part explain these associations. In this paper we determine whether use of formal preparation resources is associated with higher UCAT scores and whether differences in use of preparation resources exist between socio-demographic groups.

**Methods:**

After completing the 2017 UCAT UK school-leaver candidates (*n* = 14,332) were asked to answer a questionnaire regarding their use of official UCAT and commercial resources, school-based support, and time spent preparing. Multiple linear and logistic regression models were used to evaluate the associations between preparedness, demographic characteristics and UCAT performance.

**Results:**

Five thousand, four hundred thirty-nine (38%) candidates responded to the questionnaire. Use of freely available UCAT official practice tests, paid commercial materials, attendance at school-based preparation courses and spending more time preparing were significantly associated with higher UCAT scores. Candidates who were from less deprived backgrounds and attending independent or grammar schools were significantly more likely to use paid commercial materials and spend longer preparing.

**Conclusions:**

Reported use of preparation resources varies between candidates from different socio-demographic backgrounds and is associated independently with performance in the UCAT. Increasing the availability of freely available resources may mitigate some of these differences.

**Supplementary Information:**

The online version contains supplementary material available at 10.1186/s12909-022-03811-y.

## Introduction

There is growing use of psychometric aptitude tests by universities to assist in the selection of future medical students. Use of these tests enables medical schools to assess candidate attitudes and potential future professional behaviour, and also overcomes two existing challenges in assessing cognitive ability: (i) continued grade inflation in school-leaving examinations [1] (which historically have formed the basis for medical schools to assess candidate academic ability) which limits attempts to differentiate between applicants, and (ii) the socio-demographic patterning of performance in these examinations and which may hinder institutional efforts to widen access to medicine [2, 3].

The most widely used aptitude test in the UK is the University Clinical Aptitude Test (UCAT; previously the UK Clinical Aptitude Test (UKCAT)). Introduced in 2006 it is now used by the majority of UK medical schools as part of their student selection processes. The UCAT is a computer-based test and is delivered on behalf of the UCAT Consortium by Pearson VUE at designated test centres throughout the UK. Applicants to the medical schools using the UCAT sit the test before they make their application to medical school and can only make one attempt at the test in each annual university application cycle [4].

The UCAT comprises four cognitive subsections (verbal reasoning, quantitative reasoning, abstract reasoning, and decision making) and situational judgement test (SJT) [4]. Each cognitive section is scored separately on a scale ranging from a minimum of 300 to a maximum of 900, resulting in a total score ranging from 1200 to 3600. The SJT component is marked separately with students being placed into four Bands in which Band 1 is the best performance. Details of the distribution of total scores by decile and the proportion of students in each SJT Band for test cycles 2014–2017 are shown in Tables 1 and 2 [5].

**Table 1:** Deciles of total UKCAT score in 2014, 2015 and 2017 (NB. 2016 is excluded as the decision making section was not used operationally that year) [5]

*Decile*	2017	2015	2014
1st	2230	2210	2180
2nd	2340	2330	2310
3rd	2420	2410	2380
4th	2480	2470	2450
5th (mean score)	2540	2540	2510
6th	2600	2600	2570
7th	2670	2660	2630
8th	2750	2740	2710
9th	2860	2840	2820

**Table 2:** Proportion of candidates in each SJT Band in 2015, 2016 and 2017[5]

	**Band 1**	**Band 2**	**Band 3**	**Band 4**
2017	28%	42%	21%	9%
2016	26%	44%	22%	9%
2015	24%	45%	22%	9%

There is no consensus as to how medical schools should use the UCAT in selection and a variety of approaches exist whereby UCAT scores are used separately or integrated with other metrics to shortlist candidates for interview and/or to determine whether an offer is made [6].

Although the UCAT uses constructs which are designed to be less affected by socio-demographic factors than historical predictors of academic ability [4], studies have suggested that the inherent biases associated with the school examinations taken by UK school leavers (pupils from White and professional social-class backgrounds and attending independent or academically selective (‘grammar’) schools tend to do best) also exist for UCAT performance albeit the biases may be reduced [6]. Further, males have been shown to perform better than females on the cognitive components of the UCAT [2, 7–11] with the reverse noted for the SJT element [10, 11].

The reasons for this socio-demographic patterning of performance in the UCAT are not completely clear. We have previously suggested that access to preparation support and materials may in part explain the associations with measures of social advantage [6]. The UCAT Consortium provides free-to-use preparation resources on their website; these include practice tests, practice questions for each subsection, a candidate preparation toolkit and an official guide to the test [4]. Schools, aware that the UCAT is a key element in selection for medical degree programmes, may also include advice and practice for the UCAT as part of wider measures they have in place to support pupils to make successful applications [9]. UCAT candidates also have access to an increasing number of commercially-produced practice materials and courses which provide participants with test-taking strategies and access to a large, highly realistic bank of questions typically at a significant cost [8, 9]. Estimates concerning the use of commercial courses or materials vary with one study reporting that 20% of students attended a fee-paying course [9] whilst other studies in Australia, where a similar admissions test was used, suggest a prevalence of over 50% [12, 13].

In this paper we report a study in which we linked candidates’ responses to a questionnaire-based survey designed to elicit information on their preparation to their performance in the 2017 sitting of the UCAT. Specifically we sought to determine whether (i) formal preparation, including use of free and paid-for preparation resources, is associated with higher UCAT scores, and (ii) the demographic characteristics of those using preparation resources and whether use of preparation resources differs between socio-demographic subgroups. We limited our analyses to candidates aged 16–20 years because we wished to focus on the experience of school pupils and recent school leavers rather than graduates.

## Methods

All UK-resident candidates who sat the UCAT in 2017 were invited to participate in the study. Candidates aged under 16 years or who were aged 21 years and older were subsequently excluded from the study population to leave a sub-population we termed ‘school leavers’ (aged 16–20 years). Upon completion of the test candidates were directed to a brief on-screen questionnaire which was preceded by a statement regarding the research and a tick-box in order to gain consent. The questionnaire was created by the research team, informed by previous literature [8, 9] and comprised five questions regarding candidates’ engagement with school/college-based support, use of the official UCAT preparation materials, use of free-to-access and of paid online commercial materials, and an estimate of total time spent preparing for the test (see Additional file 1: Appendix 1). Whilst we acknowledge that books are a commonly used preparation resource, we believed these were unlikely to be used as a candidate’s primary resource. As such, the survey focused on online materials which are typically more up-to-date and reflective of the current test content and layout. Candidates were able to decline to participate at any point and could exit the UCAT without prejudice.

### Construction of dataset

When candidates complete their online registration form to secure a place to sit the UCAT they provide the following demographic information: date of birth, gender, ethnicity, parental occupation, nationality, and school/college details. Additionally, they are invited to consent to the use of this personal information and data relating to their UCAT performance by researchers to build further the evidence-based underpinning use of the test in selection. The demographic, test performance and preparation survey data of all candidates consenting to participate in research were linked to create a pseudo-anonymised dataset which was made available to the research team via the University of Dundee Health Informatics Centre’s safe haven.

### Data collection

#### Socio-demographic characteristics

Candidate age was calculated as on September 1 2018 using reported date of birth. Ethnicity was self-defined using the full Census classification and then categorised before release to the research team as Asian, Black, White, Mixed or Other. Candidates selected their school type from a drop-down list as either: Sixth Form or Further Education College, Comprehensive, Independent or Fee Paying Private, Grammar or Other. Information on parental occupation was used to derive the National Statistics Socio-economic Classification (NS-SEC) for each student in accordance with the Government method of calculation [14]. The NS-SEC is a measure of socioeconomic status with a scale ranging from 1 to 5; 1 is the least deprivation whilst 5 is the most deprived. Additionally, information was available on whether a candidate had been awarded a bursary by UCAT (candidates in financial need who meet specific criteria are eligible for a bursary covering the full test fee).

#### UCAT performance

Individual sub-section and total UCAT scores were provided for each candidate.

#### Previous attempts at the UCAT

It is possible that some candidates choose to have a ‘practice run’ at the UCAT, sitting the test in the year prior to making their application for medical school, so as to familiarize themselves with the process. This approach may be considered a form of preparation and we recorded the number of times, if any, the candidate had taken the test previously.

#### Self-reported preparation methods and time

Using the responses to the questionnaire six binary categories of preparedness were created (see Additional file 1: Appendix 2 and Table 3). It was not deemed appropriate to combine these to create a single variable to indicate if students were prepared or unprepared.

**Table 3:** Preparedness categories and how they were derived

Preparedness Category	Derivation Method
School-based preparation course	Reported having specific preparation sessions at school or college
Official UCAT Tests	Reported using the official UCAT Timed Practice Tests
Other official UCAT resources	Reported using any of the: UCAT official app, official guide or question tutorials in the Candidate Preparation Toolkit
Free commercial materials	Reported using any free commercial preparation materials
Paid commercial materials	Reported using any paid commercial preparation materials
Attempt number > 1	Having a UCAT attempt number of 2 or more
Time spent preparing	Coded according to their response to question 5 on a scale from ‘Did not prepare’ to ‘40 + hours’ of preparation

### Statistical analyses

Using a conservative estimate of a standard deviation of 300 in total UCAT score (based on UCAT data [5]), to detect a difference in UCAT score between preparedness groups of only 40 points, 1463 participants are required in each group (2926 in total) for 95% power and 5% significance. For reference, a difference of 80 points equates to moving from the 5^th^ to 6^th^ decile rank in total UCAT scores. The data collected provides over 5000 participants meaning the study is adequately powered.

All statistical analysis was conducted using Stata 15 (StataCorp LP, College Station, TX, USA). Descriptive statistics of the candidate demographics and their UCAT results were reported in terms of responders and non-responders. Chi-squared tests were conducted for the categorical and binary variables whilst independent t-tests and Mann Whitney U tests were conducted for the continuous variables, as appropriate.

In order to determine (and confirm previously described) associations between the demographic variables and UCAT performance, a multiple linear regression model was constructed with UCAT score as the outcome and gender, ethnicity, NS-SEC, school type, bursary status and attempt number as covariates. Inclusion in this aspect of the analysis was not dependent on responding to the questionnaire as all data used were routinely collected by the UCAT Consortium. A binary logistic regression model was fitted with SJT Band 1 or 2 (compared to SJT Band 3 or 4) as the outcome and the same covariates as above.

A multiple linear regression model was then fitted to investigate the association between total UCAT score (outcome variable) and each of the preparation categories, using the same covariates as above. Logistic models were fitted to investigate the association between the preparedness categories and performing well or badly on the SJT component of the test (SJT Band 1 or 2 (better performance) compared to SJT Band 3 or 4 (poorer performance) with the same covariates as above.

Binary logistic regression models were constructed, using each of the preparedness categories as an outcome and participant characteristics as covariates. Based on UCAT guidance, preparation time was categorised into those who had reported spending more than 20 h and those who had reported spending less than this.

Whilst *p*-values < 0.05 are often considered statistically significant, we would urge the reader to exercise caution when interpreting the results of the significance tests given the number of tests conducted and the resulting increased risk of type 1 error; we also encourage readers to look at the magnitude of differences between groups with corresponding confidence intervals given the large sample size.

### Research ethics statement

Ethical approval for this study was provided by the University of Birmingham Ethics Committee (*ERN_17-0521*).

## Results

**Fig. 1:**
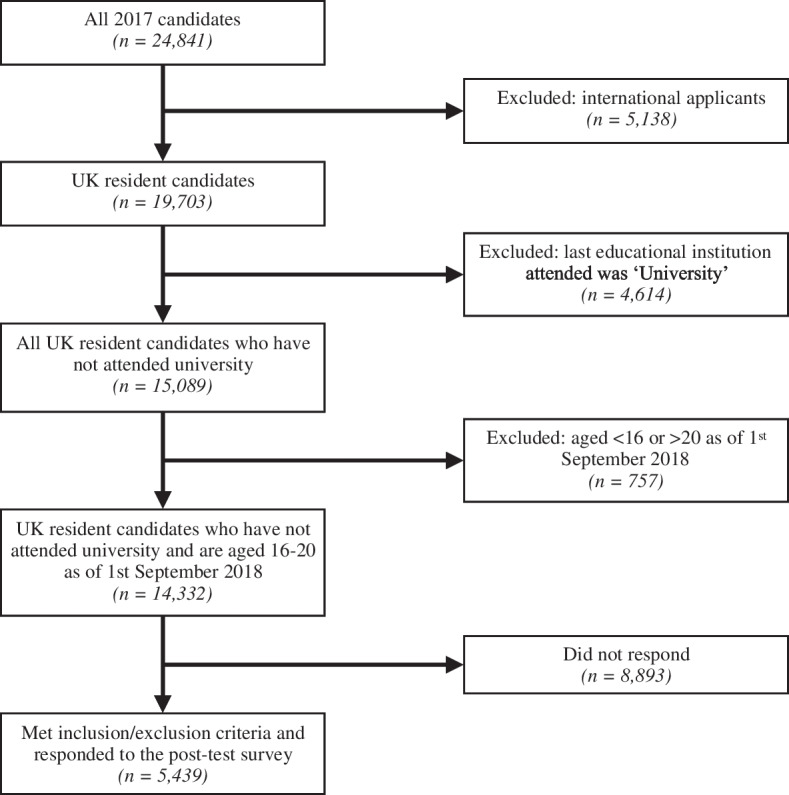
Derivation of study sample

In 2017 14,332 UK-resident candidates aged 16–20 years sat the UCAT of whom 5,439 (38%) responded to the preparedness questionnaire (Fig. 1).

### Respondent Characteristics and Preparation

Compared to non-responders, respondents were more likely to be younger, female, of White ethnicity, of higher socioeconomic status, have attended a sixth form/further education college or comprehensive school, and were less likely to have received a bursary for the test (Table 4). Although statistically significant, the absolute magnitude of differences in socio-demographic characteristics between responders and non-responders was small, due to the large sample size. For instance, the mean difference in age between responders and non-responders, whilst statistically significant, was 18 days. Responders also performed better than non-responders in the test overall, and in each subsection, with the exception of the abstract reasoning component (Table 5). A significantly greater proportion of respondents achieved an SJT Band 1 or 2 compared to Band 3 or 4 (*p* = 0.025) and were taking the test for the first time.

**Table 4:** Demographic characteristics of the study population in terms of responders and non-responders

	**Responders** *(n* = *5439 (38.0))* **N (%)**	**Non-responders** *(n* = *8893 (62.1))* **N (%)**	**All** *(n* = *14,432)* **N (%)**	***p*-values**
**Mean age (SD)**	18.66 (0.59)	18.71 (0.61)	18.69 (0.60)	< *0.001*
**Gender**				< *0.001*
**Male**	1967 (36.2)	3494 (39.3)	5461 (38.1)	
**Female**	3472 (63.8)	5399 (60.7)	8871 (61.9)	
**Ethnicity**				< *0.001*
*White*	2609 (48.0)	3851 (43.3)	6460 (45.1)	
*Asian*	1816 (33.4)	3505 (39.4)	5321 (37.1)	
*Black*	521 (9.6)	670 (7.5)	1191 (8.3)	
*Mixed*	288 (5.3)	441(5.0)	729 (5.1)	
*Other*	205 (3.8)	426 (4.8)	631 (4.4)	
**NS-SEC**				*0.024*
*1*	4160 (76.5)	6543 (73.6)	10,703 (74.7)	
*2*	213 (3.9)	302 (3.4)	515 (3.6)	
*3*	80 (1.5)	182 (2.1)	262 (1.8)	
*4*	212 (3.9)	381 (4.3)	593 (4.1)	
*5*	364 (6.7)	580 (6.5)	944 (6.6)	
*Missing*	410 (7.5)	905 (10.2)	1315 (9.2)	
**SCHOOL**				< *0.001*
*Sixth Form/ Further Education College*	2689 (49.4)	4376 (49.2)	7065 (49.3)	
*Comprehensive*	705 (13.0)	944 (10.6)	1649 (11.5)	
*Grammar*	1006 (18.5)	1843 (20.7)	2849 (19.9)	
*Independent/Private Fee Paying*	958 (17.6)	1636 (18.4)	2594 (18.1)	
*Other*	80 (1.5)	94 (1.1)	174 (1.2)	
*Missing*	1 (0.0)	0 (0.0)	1 (0.0)	
**BURSARY**				*0.014*
**Bursary received**	584 (10.7)	1075 (12.1)	1659 (11.6)	
**No bursary received**	4855 (89.3)	7818 (87.9)	12,673 (88.4)	

*P*-values for the chi-squared tests are presented except for age where an independent t-test was conducted. Unless stated, there was no missing data for the variables. Missing data was not used in chi-squared calculations

**Table 5:** UCAT total and sub-section scores for responders and non-responders

	**Responders**	**Non-responders**	**All**	***p*-values**
**Mean total UCAT score (SD)**	2590 (243)	2566 (230)	2575 (235)	< *0.001*
**Mean abstract reasoning score (SD)**	639 (85.4)	638 (82.1)	638 (83.4)	*0.372*
**Mean verbal reasoning score (SD)**	583 (79.1)	571 (74.5)	575 (76.5)	< *0.001*
**Mean decision making score (SD)**	658 (54.0)	651 (52.8)	654 (53.4)	< *0.001*
**Mean quantitative reasoning score (SD)**	710 (92.9)	706 (90.7)	707 (91.6)	*0.003*
**SJT Band** **(N (%))**				*0.025*
*1 or 2*	4057 (74.6)	6482 (72.9)	10,539 (73.5)	
*3 or 4*	1382 (25.4)	2411 (27.1)	3793 (26.5)	
**UCAT Attempt Number (N (%))**				< *0.001*
*1st*	5056 (93.0)	8072 (90.8)	13,128 (91.6)	
*2nd or more*	383 (7.0)	821 (9.2)	1204 (8.4)	
**Test Type (N (%))**				*0.419*
*UCAT*	5254 (96.6)	8542 (96.1)	13,796 (96.3)	
*UCATSEN*	178 (3.3)	338 (3.8)	516 (3.6)	
*UCATSA*	4 (0.1)	8 (0.1)	12 (0.1)	
*UCAT50*	3 (0.1)	5 (0.1)	8 (0.1)	

*P*-values are shown for the independent t-tests (for UCAT and subsection scores) and chi-squared tests (for categorical variable) were conducted to identify differences between responders and non-responders

Among responders (*n* = 5439), 877 (16.1%) reported attendance at a school-based preparation course, 4434 (81.5%) reported using the official UCAT practice tests and 3047 (56%) reported using paid commercial materials (Table 6). Only 34 (0.6%) respondents reported not spending any time preparing for the test whilst 1320 (24.3%) prepared for over 40 h (Table 7).

**Table 6:**
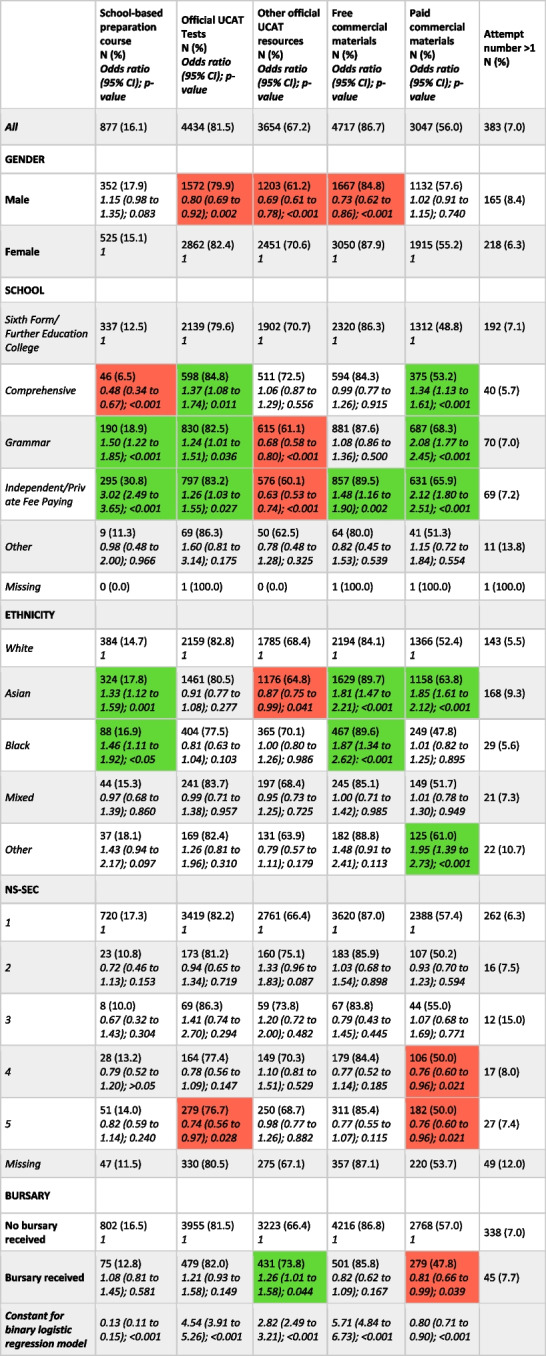
Demographic characteristics by preparedness category. Below are binary logistic regression models with each of the preparedness categories (with the exception of retaking) as the outcome variables and demographic variables as covariates. The reference category for each variable has an odds ratio of 1. Significant results with negative direction of effect have been shaded red whilst significant results with positive direction of effect have been shaded green

Please note that only candidates with non-missing values were included within the regression analysis

**Table 7:**
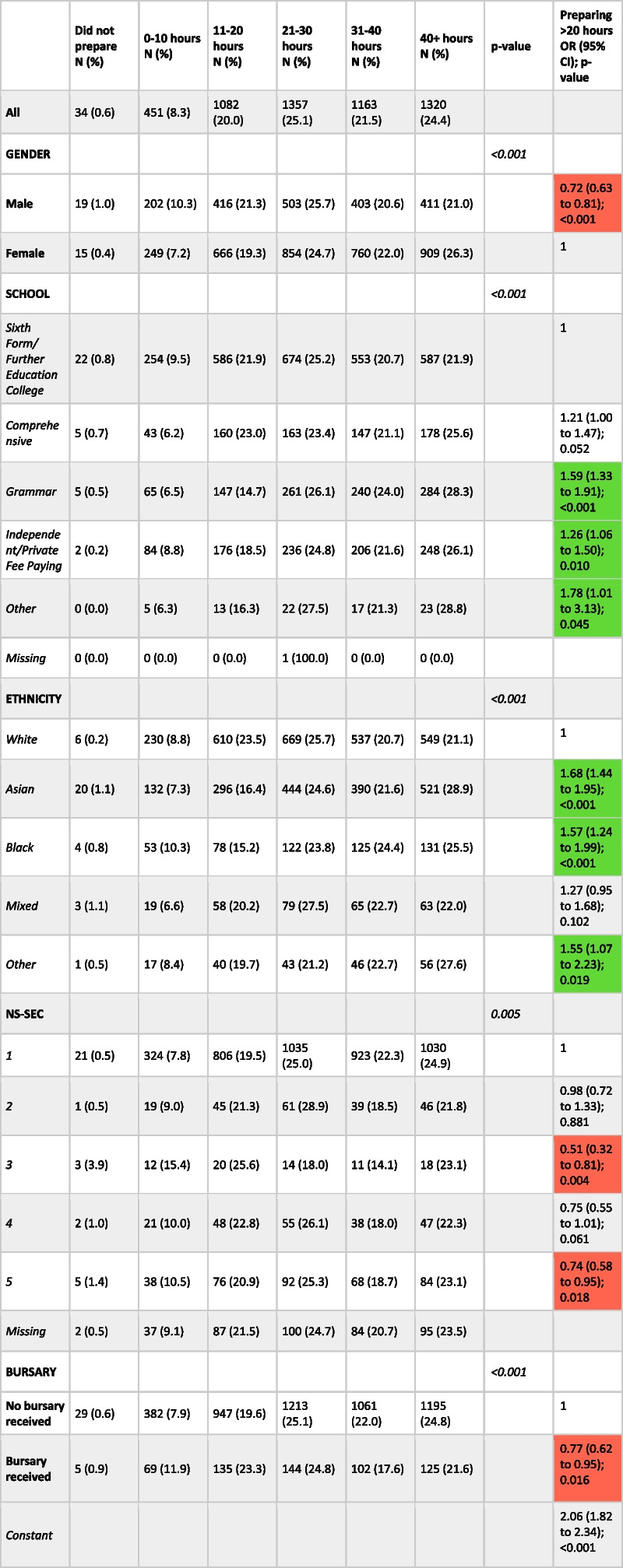
Demographic characteristics by time spent preparing for the test. Binary logistic regression model with preparing > 20 h as the outcome variable and demographic variables as covariates as the final column. The reference category for each variable has an odds ratio of 1. Missing values not included in chi-squared tests or regression analysis. Significant results with negative direction of effect have been shaded red whilst significant results with positive direction of effect have been shaded green

Note that only 5407 participants reported the time they spent preparing hence all percentages are calculated out of this value

### Socio-demographic factors and UCAT performance

The results of the multiple linear regression model, constructed using all candidates who were UK residents and aged 16–20 years and with non-missing values (*n* = 13,016), indicate that candidates who are male, attend grammar or independent or private fee-paying schools, were of White ethnicity or of higher socioeconomic background perform significantly better on the test (Table 8). Further test subsection analyses can be found in Additional file 1: Appendix 3.

**Table 8:** Multiple linear regression model with total UCAT score as the outcome and preparedness categories and demographic variables as covariates. The third and fourth columns show the multiple linear regression model with total UCAT score as the outcome and demographic variables as covariates using all included candidates (i.e. responders and non-responders) and does not therefore adjust for preparedness

Variable	Mean difference in UCAT score including preparedness variables (95% CI)	*p*-value	Mean difference in UCAT score using all eligible candidates (95% CI)	*p*-value
**Gender**				
*Female*	0		0	
*Male*	52.78 (40.27 to 65.28)	< 0.001	48.36 (40.75 to 55.96)	< 0.001
**School type**				
*Sixth Form/Further Education College*	0		0	
*Grammar*	112.57 (96.00 to 129.14)	< 0.001	112.00 (102.14 to 121.87)	< 0.001
*Independent/ Private Fee Paying*	94.21 (77.03 to 111.40)	< 0.001	100.32 (90.01 to 110.63)	< 0.001
*Comprehensive*	16.17 (-2.73 to 35.08)	0.094	27.22 (15.08 to 39.36)	< 0.001
*Other*	-47.10 (-96.64 to 2.44)	0.062	-25.56 (-59.99 to 8.88)	0.146
**Ethnicity**				
*White*	0		0	
*Asian*	-103.87 (-118.07 to -89.67)	< 0.001	-77.05 (-85.55 to -68.56)	< 0.001
*Black*	-205.29 (-227.34 to -183.24)	< 0.001	-178.50 (-192.96 to -164.04)	< 0.001
*Mixed*	-55.71 (-82.72 to -28.69)	< 0.001	-38.62 (-55.59 to -21.64)	< 0.001
*Other*	-129.34 (-163.71 to -94.98)	< 0.001	-103.81 (-123.68 to -83.93)	< 0.001
**NS-SEC**				
*1*	0		0	
*2*	-22.40 (-52.40 to 7.59)	0.143	-17.20 (-36.35 to 1.96)	0.078
*3*	-50.98 (-99.39 to -2.56)	0.039	-65.66 (-92.05 to -39.27)	< 0.001
*4*	-50.55 (-80.58 to -20.52)	0.001	-74.20 (-92.27 to -56.13)	< 0.001
*5*	-64.52 (-88.82 to -40.21)	< 0.001	-71.98 (-87.08 to -56.87)	< 0.001
**Second or more than second attempt of UCAT**	66.64 (42.69 to 90.60)	< 0.001	46.77 (33.07 to 60.47)	< 0.001
**School-based preparation course**	36.66 (20.25 to 53.07)	< 0.001		
**Official UCAT Tests**	67.77 (52.01 to 83.53)	< 0.001		
**Other official UCAT resources**	-1.24 (-14.12 to 11.65)	0.851		
**Free commercial materials**	-13.95 (-31.88 to 3.99)	0.127		
**Paid commercial materials**	37.65 (24.81 to 50.49)	< 0.001		
**Time preparing**				
*Did not prepare*	0			
*0–10 h*	68.48 (-8.60 to 145.56)	0.082		
*11–20 h*	124.02 (48.37 to 199.67)	0.001		
*21–30 h*	131.75 (56.21 to 207.29)	0.001		
*31–40 h*	165.88 (90.06 to 241.71)	< 0.001		
*40* + *hours*	177.90 (102.12 to 253.69)	< 0.001		
**Bursary**				
*No bursary received*				
*Bursary received*	-12.12 (-33.51 to 9.26)	0.266	-19.37 (-32.50 to -6.24)	0.004
*Constant*	2390.72 (2313.75 to 2467.68)	< 0.001	2577.32 (2569.56 to 2585.08)	< 0.001

The first model used all eligible responders with non-missing values (*n* = 5001). For each of the preparedness categories, the reference category is not being in the category. For example, for ‘paid preparation materials’, the reference category is those who did not report using paid preparation materials

A binary logistic regression model (Table 9), with the Band 1 or 2 (better performance) in the SJT component as the outcome (compared to Band 3 or 4), showed that males were significantly less likely to achieve the top two Bands (Band 1 or 2) than females. Those from independent, grammar and comprehensive schools were significantly more likely to be placed in the top two Bands compared to those attending a sixth form or college. Candidates of Asian and Black ethnicities performed significantly worse than those of White ethnicity. Candidates from more deprived backgrounds performed significantly worse than those from least deprived backgrounds. Students who were retaking the test were significantly more likely to obtain a Band 1 or 2; further analysis with Band 4 as the outcome variable showed similar results (Additional file 1: Appendix 4).

**Table 9:** Binary logistic regression model with SJT Band 1 or 2 as the outcome using all included candidates and does not adjust for preparedness (*n* = 14,432). Binary logistic regression model with SJT Band 1 or 2 as the outcome and preparedness categories and demographic variables as covariates using all responder (*n* = 5439)

Variable	Adjusted odds ratio (95% CI)	*p*-value	Adjusted odds ratio using all eligible candidates (95% CI)	*p*-value
**Gender**				
*Female*	1		1	
*Male*	0.65 (0.57 to 0.75)	< 0.001	0.62 (0.57 to 0.68)	< 0.001
**School type**				
*Sixth Form/Further Education College*	1		1	
*Grammar*	1.32 (1.09 to 1.59)	0.004	1.59 (1.42 to 1.78)	< 0.001
*Independent/ Private Fee Paying*	1.53 (1.24 to 1.88)	< 0.001	1.53 (1.36 to 1.72)	< 0.001
*Comprehensive*	1.19 (0.95 to 1.48)	0.124	1.22 (1.07 to 1.40)	0.004
*Other*	0.74 (0.43–1.27)	0.274	0.86 (0.60 to 1.25)	0.438
**Ethnicity**				
*White*	1		1	
*Asian*	0.46 (0.39 to 0.54)	< 0.001	0.55 (0.50 to 0.60)	< 0.001
*Black*	0.37 (0.29 to 0.47)	< 0.001	0.45 (0.39 to 0.53)	< 0.001
*Mixed*	0.64 (0.47 to 0.88)	0.005	0.82 (0.68 to 1.00)	0.047
*Other*	0.40 (0.28 to 0.57)	< 0.001	0.58 (0.47 to 0.72)	< 0.001
**NS-SEC**				
*1*	1		1	
*2*	1.01 (0.71 to 1.41)	0.976	1.03 (0.83 to 1.27)	0.811
*3*	0.94 (0.55 to 1.58)	0.802	0.73 (0.56 to 0.95)	0.021
*4*	0.73 (0.54 to 1.00)	0.047	0.68 (0.56 to 0.81)	< 0.001
*5*	0.67 (0.52to 0.85)	0.001	0.74 (0.63 to 0.86)	< 0.001
**Second or more than second attempt of UCAT**	2.28 (1.66 to 3.13)	< 0.001	2.23 (1.86 to 2.67)	< 0.001
**School-based preparation course**	1.40 (1.15 to 1.71)	0.001		
**Official UCAT Tests**	1.15 (0.97 to 1.37)	0.110		
**Other official UCAT resources**	0.94 (0.81 to 1.09)	0.401		
**Free commercial materials**	1.04 (0.85 to 1.26)	0.729		
**Paid commercial materials**	1.15 (1.00 to 1.33)	0.055		
**Time preparing**				
*Did not prepare*	1			
*0–10 h*	1.28 (0.61 to 2.71)	0.516		
*11–20 h*	2.07 (0.99 to 4.34)	0.052		
*21–30 h*	2.28 (1.09 to 4.76)	0.028		
*31–40 h*	3.64 (1.73 to 7.66)	0.001		
*40* + *hours*	3.64 (1.73 to 7.64)	0.001		
**Bursary**				
*No bursary received*	1		1	
*Bursary received*	1.23 (0.98 to 1.56)	0.078	1.05 (0.92 to 1.21)	0.468
*Constant*	1.48 (0.70 to 3.16)	0.308	4.10 (3.75 to 4.47)	< 0.001

For each of the preparedness categories, the reference category is not being in the category. For example, for ‘paid preparation materials’, the reference category is those who did not report using paid preparation materials

### Impact of preparation on UCAT performance

**Fig. 2:**
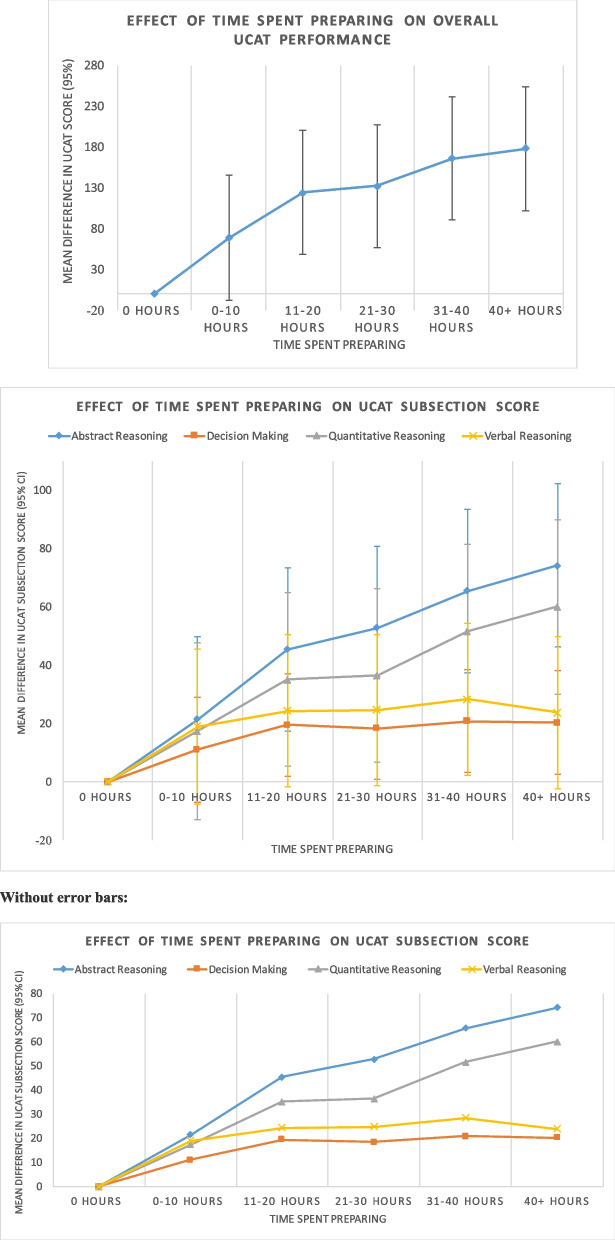
Graphs of time spent preparing compared to UCAT performance both overall and in each subsection. The data has been generated from the multiple logistic regression models outlined in the methods and results with error bars relating to the 95% confidence interval for each data point. The mean difference in UCAT score for each category is compared to the average score for the ‘Did not prepare category (i.e. 0 h)

Use of the freely available official UCAT timed practice tests was associated with greatest impact on test performance (MD = 67.77, 95% CI: 52.01 to 83.53; *p* < 0.001) (Table 8). Attendance at a school-based preparation course (MD = 36.66, 95% CI: 20.25 to 53.07) or using paid commercial materials (MD = 37.65, 95% CI: 24.81 to 50.49) also significantly improve test performance (*p* < 0.001 for both). Overall UCAT performance improved as the time spent preparing increased (see Fig. 2). When evaluating subsections of the UCAT, performance appeared to increase with greater preparation time categories for the abstract reasoning and quantitative reasoning subsections only; for other subsections performance seems to plateau at moderate levels of preparation. Differences in scores between those who retook the test, used paid commercial materials or spent longer preparing, compared to those who did not, were largely observed in the abstract reasoning and quantitative reasoning subsections (Additional file 1: Appendix 5). Attendance at a school-based preparation course and studying for over 20 h for the test were both found to be significantly associated with achieving a Band 1 or 2 in the SJT (Table 9). Moreover, the socio-demographic patterns in UCAT performance seen using all eligible candidates such as better performance by males or those of White ethnicity, remain despite adjustment for preparedness in our model.

### Use of preparation materials and time taken to prepare, and candidate characteristics

Males were significantly less likely to report using the official UCAT practice tests, using free commercial materials or to prepare for more than 20 h for the test than females (see Tables 6 and 7). Compared to those at sixth form or college, students attending grammar and independent schools were significantly more likely to report attendance at a school-based preparation course with odds ratios of 1.50 (95% CI: 1.22 to 1.85, *p* < 0.001) and 3.02 (95% CI: 2.49 to 3.65, *p* < 0.001) respectively. Similarly, those attending independent or grammar schools were significantly more likely to use the official UCAT tests, paid commercial and to prepare for more than 20 h for the test compared to those attending sixth forms or colleges.

Students of Asian or Black ethnicity were significantly more likely to have reported attending a school-based preparation course, using free commercial materials or spending more than 20 h preparing for the test compared to those of White ethnicity. Compared to White students, those of Asian ethnicity were significantly more likely to use paid commercial resources.

Candidates in most socio-economically deprived quintile were significantly less likely to use the official tests, use paid commercial materials or prepare more than 20 h for the test compared to the least deprived (those with an NS-SEC of 1). Students in receipt of a bursary were significantly less likely to report using paid commercial materials or to prepare for more than 20 h for the test compared to those who had not received a bursary.

## Discussion

Our analysis indicates that use of the official UCAT materials, use of paid commercial materials, attendance at school-based preparation courses and spending more time preparing are all associated with better performance on the cognitive components of the test. A dose–response relationship was observed for the increasing time spent preparing with no apparent ceiling of effect. Statistically significant differences in the use of these preparation resources were identified between different socio-demographic groups and those from differing school types.

With regards to the SJT component, spending more time preparing for the test was a significant predictor of better performance but the findings indicate no evidence of an association between use of specific preparation resources and performance with the exception of attendance at school-based preparation courses. One explanation for this finding may be that because SJT performance correlates with personality traits such as conscientiousness [15, 16], those spending longer preparing for the test are more likely to be conscientious candidates and will therefore inherently perform better on this component.

Our findings reiterate the socio-demographic patterning reported in UCAT and SJT performance by otherss [2, 6–8, 10, 11]. We note also that candidates in receipt of UCAT Consortium bursaries perform significantly worse on the test.

Our data suggest that candidates using preparation resources perform better in the test, an observation which concurs with the literature on the Undergraduate Medicine and Health Sciences Admissions Test (UMAT) used in Australia [12, 13]. Two questionnaire-based studies by Lambe et al. have previously investigated the impact of modes of preparation on self-reported UCAT performance [8, 9]. The first study found that increased preparation time correlated with higher test scores [8] whilst the second noted that those who used UCAT-provided practice tests performed 67 points better than those who did not (95% CI: 22.6 to 110.3, *p* < 0.001) [9]. Further interpretation of these observations is compromised by absence of information on socio-demographics of respondents and non-respondents [8, 9]. In contrast to our study, Lambe et al. did not identify a significant difference in UCAT performance between those who had attended school or commercial courses and those who had not. However the reported use of paid commercial coaching resources and school-based preparation courses by their participants was markedly lower than that reported in our study (9% commercial and 7% school-based versus 56% and 16% respectively) [8, 9].

Our analysis suggests that the differences in UCAT scores associated with preparedness can largely be attributed to better performance in the abstract reasoning and quantitative reasoning subsections which appears to echo the findings from the equivalent sections of the UMAT [12, 13]. In our subsection analysis, only performance in these sections demonstrated a dose–response relationship with time spent preparing. The researchers investigating the UMAT argued that these sections are more easily answered using learned, specific problem-solving skills whilst the verbal reasoning component is less amenable to using learned techniques from coaching [12].

An important limitation of this study is the likelihood of responder bias. Whilst the responders and non-responders were largely comparable in terms of whole numbers with regards to demographic characteristics, those who responded performed significantly better on the test. Although responders were unaware of their test score when completing the survey, it is possible that those who believed they had not performed well were less likely to complete our questionnaire. We also acknowledge the categories used for some of the variables, such as ethnicity and school type, are very broad and as a result there is likely to be a degree of variability within each group. An additional limitation is the lack of a measure of prior academic attainment for each candidate, which is a known predictor of UCAT performance [2] and may possibly moderate the impact of preparation [13]. In the future we plan to combine our data with the UKMED dataset to allow adjustment for further confounders such as academic attainment and to answer further research questions such as the impact of preparation on the predictive validity of the UCAT. Similarly, in the absence of a measure of pre-preparation UCAT score it is not possible to determine at an individual level whether preparation leads to an improvement in test performance.

We further acknowledge that there is likely a degree of correlation within the different demographic and preparedness variables, for instance between school type and socioeconomic status or preparation time and use of commercial materials. These inter-variable relationships may influence the independent effect of each variable and further research to delineate this would be useful (although was considered beyond the scope of this paper). In addition, qualitative research investigating candidate preparation in relation to socio-demographic characteristics and social norms may further our understanding of the issues.

The effectiveness of preparation for cognitive tests may be attributed to improving candidates’ test taking techniques and pattern recognition skills but perhaps also by increasing their familiarity with the test format and various question types. Medical schools should be cautious about presuming these types of selection tests only assess inherent intellectual ability. It is possible that access to preparation tools and support may partially explain the association between higher UCAT scores and attendance at a selective schools and higher socioeconomic family background. The key implication, therefore, is that addressing the differential in access to preparation resources may mitigate differences in performance between socio-demographic groups. Suggested means of achieving this include creating more freely available resources through the UCAT website and increased awareness of such resources.

Preparation was found to have little association with performance in the SJT component, yet differences of a large magnitude exist between ethnic and socioeconomic groups; language, cultural and social norms may be important determinants of SJT performance, rather than preparedness.

## Conclusions

This study has demonstrated an association between use of preparatory materials and courses and scores in the 2017 UCAT and demonstrated differential use of preparation resources among candidates from different socio-demographic groups. We have also confirmed previous reports of associations between candidate performance and demographic characteristics including ethnicity and family socio-economic status.

## Supplementary Information

**Additional file 1: Appendix 1.** UCAT Post-test Questionnaire. **Appendix 2.** Questionnaire Responses. **Appendix 3.** Subsection score regression models with demographic variables as covariates. **Appendix 4.** SJT Band 4 binary logistic regression models with demographic variables as covariates. **Appendix 5.** Subsection score regression models with demographic variables and preparedness categories as covariates.

## Data Availability

The data that support the findings of this study are available from the University Clinical Aptitude Test Consortium but restrictions apply to the availability of these raw data due to confidentiality assurances provided to UCAT test-takers (see: Candidate Data | UCAT Consortium). Data are available from Jayne Parry upon reasonable request and with permission of the University Clinical Aptitude Test.

## References

[1] Patterson F, Knight A, Dowell J, Nicholson S, Cousans F, Cleland J (2016). How effective are selection methods in medical education? A systematic review. Med Educ..

[2] Tiffin PA, McLachlan JC, Webster L, Nicholson S (2014). Comparison of the sensitivity of the UKCAT and A Levels to sociodemographic characteristics: a national study. BMC Med Educ..

[3] Department for Education. Revised A level and other 16–18 results in England, 2016/2017. 2018; https://assets.publishing.service.gov.uk/government/uploads/system/uploads/attachment_data/file/676389/SFR03_2018_Main_text.pdf. Accessed 15 Nov 2018.

[4] UKCAT. UKCAT Test. 2017; https://www.ukcat.ac.uk/ukcat-test/. Accessed 15 Nov 2018.

[5] UKCAT. UKCAT Test Statistics 2016 - 2017. 2018; https://www.ukcat.ac.uk/media/1267/ukcat-test-statistics-2016_2017.pdf. Accessed 17 Nov 2018.

[6] Mathers J, Sitch A, Parry J (2016). Longitudinal assessment of the impact of the use of the UK clinical aptitude test for medical student selection. Med Educ.

[7] James D, Yates J, Nicholson S (2010). Comparison of A level and UKCAT performance in students applying to UK medical and dental schools in 2006: cohort study. Bmj..

[8] Lambe P, Waters C, Bristow D (2012). The UK Clinical Aptitude Test: is it a fair test for selecting medical students?. Med Teach.

[9] Lambe P, Greatrix R, Milburn K, Dowell J, Bristow D (2016). Do differentials in access to advice and support at UK schools on preparation for the UK Clinical Aptitude Test disadvantage some candidate groups?.

[10] Lievens F, Patterson F, Corstjens J, Martin S, Nicholson S (2016). Widening access in selection using situational judgement tests: evidence from the UKCAT. Med Educ.

[11] Husbands A, Dowell J, Homer M, McAndrew R, Greatrix R (2018). Exploring the Relationship between the UKCAT Situational Judgement Test and the Multiple Mini Interview.

[12] Griffin B, Harding DW, Wilson IG, Yeomans ND (2008). Does practice make perfect? The effect of coaching and retesting on selection tests used for admission to an Australian medical school. Med J Aust.

[13] Griffin B, Carless S, Wilson I (2013). The effect of commercial coaching on selection test performance. Med Teach.

[14] Office for National Statistics. The National Statistics Socio-economic Classification: (Rebased on the SOC2010) User Manual. 2010; https://www.ons.gov.uk/methodology/classificationsandstandards/standardoccupationalclassificationsoc/soc2010/soc2010volume3thenationalstatisticssocioeconomicclassificationnssecrebasedonsoc2010. Accessed 25 Jan 2019.

[15] Tiffin PA, Patton LW. Exploring the validity of the 2013 UKCAT SJT- prediction of undergraduate performance in the first year of medical school: Summary Version of Report. 2017; https://www.ucat.ac.uk/media/1119/exploring-the-validity-of-the-2013-ukcat-sjt-prediction-of-ug-performance-in-1st-yr-of-med-school-summary-version-posted-27032017.pdf. Accessed 28 Aug 2020.

[16] Husbands A, Rodgerson MJ, Dowell J, Patterson F (2015). Evaluating the validity of an integrity-based situational judgement test for medical school admissions. BMC Med Educ..

