# Supplementary material for: An assessment of the impact of formal preparation activities on performance in the University Clinical Aptitude Test (UCAT): a national study
Source: BMC Med Educ. 2022 Oct 28;22:747. doi: 10.1186/s12909-022-03811-y (PMC9617356; doi:10.1186/s12909-022-03811-y)
Supplement: Supplementary file 1 — Additional file 1: Appendix 1. UCAT Post-test Questionnaire. Appendix 2. Questionnaire Responses. Appendix 3. Subsection score regression models with demographic variables as covariates. Appendix 4. SJT Band 4 binary logistic regression models with demographic variables as covariates. Appendix 5. Subsection score regression models with demographic variables and preparedness categories as covariates. [file 12909_2022_3811_MOESM1_ESM.docx]

**An assessment of the impact of formal preparation activities on performance in the University Clinical Aptitude Test (UCAT): a national study.**

**Supplementary Materials**

# Appendices

1. Appendix 1: UCAT Post-test Questionnaire
2. Appendix 2: Questionnaire Responses
3. Appendix 3: Subsection score regression models with demographic variables as covariates
4. Appendix 4: SJT Band 4 binary logistic regression models with demographic variables as covariates
5. Appendix 5: Subsection score regression models with demographic variables and preparedness categories as covariates

## Appendix 1: UCAT Post-test Questionnaire

**COPY OF THE PREPAREDNESS QUESTIONS AND PREFACE APPENDED TO THE UCAT EXAMINATION 2017**

**Post Test Survey – Preparing for the UCAT**

UCAT is interested to find out how candidates prepare to take the test and to investigate how preparation might impact on test performance.  The results from this survey will be used to inform the future development of freely available preparation materials provided by UCAT.

Data collected from this survey will be used for research purposes and combined with your test data and demographic data obtained from you at registration.  Researchers will have access to anonymised data only and no individuals will be identifiable in subsequent publications.

Please tick here if you are happy to continue with this short survey and agree to this data to be used in this way.

1. What advice has your school/college given you on preparation for the UCAT? Tick all that apply.

- No advice given
- My school/college directed me to the UCAT website
- My school/college directed me to other preparation materials
- My school/college provided specific advice on taking the test
- My school/college arranged specific preparation sessions for the UCAT
- N/A (e.g. no longer at school/college)

1. UCAT provides a large number of **free preparation materials** on our website. Which of the following **FREE UCAT** preparation materials did you use? Tick all that apply.

- Timed Practice Tests (e.g. A, B, C)
- UCAT Official app
- Official Guide
- Question Tutorial in Candidate Preparation Toolkit

1. Some test preparation providers provide **FREE preparation materials** to assist candidates with test preparation. Did you access **FREE preparation materials** from any of these providers?  Tick all that apply.

- Kaplan
- Medic Portal
- Medify
- JobTest Prep
- UCAT help.com
- Get into Medicine
- Prepgenie
- Other please specify

1. Test preparation providers also provide **PAID for preparation materials** to candidates.  Did you access **PAID for preparation materials** from any of these providers?  Tick all that apply.

- Kaplan self study
- Kaplan live online
- Kaplan Classroom
- Medic Portal
- Medify
- 6med Crash Course
- UCAT help.com
- JobTest Prep
- Blue Peanut
- Prepgenie
- Pastest
- Emedica
- Other please specify

1. Approximately how many **hours** did you spend preparing for the UCAT?  Please include time spent on all modes of preparation (e.g. practice tests, reading, course attendance etc.).

- 0-10 hours
- 11-20 hours
- 21-30 hours
- 30-40 hours
- 40+ hours
- Did not prepare

## Appendix 2: Questionnaire Responses

The table below shows the numbers and percentages of respondents (*n*=5439) who chose each of the responses.

| **Question** | **Number who answered (%)** |
| --- | --- |
| **1. What advice has your school/college given you on preparation for the UCAT?** | |
| *No advice given* | 2439 (44.8) |
| *My school/college directed me to the UCAT website* | 1949 (35.8) |
| *My school/college directed me to other preparation materials* | 1452 (26.7) |
| *My school/college provided specific advice on taking the test* | 711 (13.1) |
| *My school/college arranged specific preparation sessions for the UCAT* | 877 (16.1) |
| *N/A (e.g. no longer at school/college)* | 159 (2.9) |
|  |  |
| **2. UCAT provides a large number of free preparation materials on our website. Which of the following FREE UCAT materials did you use?** | |
| *Timed Practice Tests (e.g. A, B, C)* | 4434 (81.5) |
| *UCAT Official app* | 2342 (43.1) |
| *Official Guide* | 1995 (36.7) |
| *Question Tutorial in Candidate Preparation Toolkit* | 1704 (31.3) |
|  |  |
| **3. Some test preparation providers provide FREE preparation materials to assist candidates with test preparation. Did you access FREE preparation materials from any of these providers?** | |
| *Kaplan* | 1393 (25.6) |
| *Medic Portal* | 3266 (60.0) |
| *Medify* | 2057 (37.8) |
| *JobTest Prep* | 424 (7.8) |
| *UCAThelp.com* | 676 (12.4) |
| *Get into Medicine* | 513 (9.4) |
| *Prepgenie* | 22 (0.4) |
| *Other* | 230 (4.2) |
|  |  |
| **4. Test preparation providers also provide PAID for preparation materials to candidates. Did you access PAID for preparation materials from any of these providers?** | |
| *Kaplan self study* | 497 (9.1) |
| *Kaplan live online* | 175 (3.2) |
| *Kaplan Classroom* | 452 (8.3) |
| *Medic Portal* | 621 (11.4) |
| *Medify* | 2001 (36.8) |
| *6Med Crash Course* | 167 (3.1) |
| *UCAThelp.com* | 66 (1.2) |
| *JobTest Prep* | 36 (0.7) |
| *Blue Peanut* | 7 (0.1) |
| *Prepgenie* | 4 (0.1) |
| *Pastest* | 8 (0.1) |
| *Emedica* | 44 (0.8) |
| *Other* | 57 (1.0) |
|  |  |
| **5. Approximately how many hours did you spend preparing for the UCAT? Please include time spent on all modes of preparation (e.g. practice tests, reading, course attendance etc.).** | |
| *0-10 hours* | 451 (8.3) |
| *11-20 hours* | 1082 (19.9) |
| *21-30 hours* | 1357 (24.9) |
| *31-40 hours* | 1163 (21.4) |
| *40+ hours* | 1320 (24.3) |
| *Did not prepare* | 34 (0.6) |

## Appendix 3: Subsection score regression models with demographic variables as covariates

The following are multiple linear regression models with each subsection score as the outcome variable and demographic variables as covariates using all eligible participants. The mean difference for each included variable in the model is equivalent to the mean difference in total UCAT score for that category compared to a reference category when adjusting for the other included covariates. The reference category for each variable has a mean difference of zero.

**Abstract Reasoning**

| **Variable** | **Mean difference (95% CI)** | **p-value** |
| --- | --- | --- |
| **GENDER** |  |  |
| *Female* | 0 |  |
| *Male* | 2.37 (-0.49 to 5.23) | 0.104 |
| **SCHOOL TYPE** |  |  |
| *Sixth Form/Further Education College* | 0 |  |
| *Grammar* | 32.82 (29.11 to 36.53) | <0.001 |
| *Independent/ Private Fee Paying* | 30.85 (26.98 to 34.73) | <0.001 |
| *Comprehensive* | 7.86 (3.30 to 12.42) | 0.001 |
| *Other* | -2.35 (-15.29 to 10.60) | 0.722 |
| **ETHNICITY** |  |  |
| *White* | 0 |  |
| *Asian* | -4.82 (-8.01 to -1.62) | 0.003 |
| *Black* | -38.03 (-43.46 to -32.60) | <0.001 |
| *Mixed* | -0.81 (-7.19 to 5.57) | 0.804 |
| *Other* | -8.78 (-16.26 to -1.31) | 0.021 |
| **NS-SEC** |  |  |
| *1* | 0 |  |
| *2* | -9.52 (-16.72 to -2.31) | 0.010 |
| *3* | -17.01 (-26.93 to -7.09) | 0.001 |
| *4* | -17.62 (-24.41 to -10.83) | <0.001 |
| *5* | -19.29 (-24.96 to -13.61) | <0.001 |
| **ATTEMPT NUMBER** |  |  |
| *First attempt of UCAT* | 0 |  |
| *Second or more than second attempt of UCAT* | 19.08 (13.93 to 24.23) | <0.001 |
| **BURSARY STATUS** |  |  |
| *No bursary received* | 0 |  |
| *Bursary received* | -7.04 (-11.98 to -2.11) | 0.005 |
| *Constant* | 633.37 (630.45 to 636.29) | <0.001 |

**Decision Making**

| **Variable** | **Mean difference (95% CI)** | **p-value** |
| --- | --- | --- |
| **GENDER** |  |  |
| *Female* | 0 |  |
| *Male* | 4.92 (3.15 to 6.69) | <0.001 |
| **SCHOOL TYPE** |  |  |
| *Sixth Form/Further Education College* | 0 |  |
| *Grammar* | 16.07 (13.77 to 18.36) | <0.001 |
| *Independent/ Private Fee Paying* | 17.50 (15.10 to 19.90) | <0.001 |
| *Comprehensive* | 1.67 (-1.16 to-4.49) | 0.247 |
| *Other* | -8.35 (-16.36 to -0.35) | 0.041 |
| **ETHNICITY** |  |  |
| *White* | 0 |  |
| *Asian* | -23.78 (-25.75 to -21.81) | <0.001 |
| *Black* | -37.51 (-40.87 to -34.15) | <0.001 |
| *Mixed* | -10.95 (-14.89 to -7.00) | <0.001 |
| *Other* | -29.54 (-34.16 to -24.92) | <0.001 |
| **NS-SEC** |  |  |
| *1* | 0 |  |
| *2* | -2.72 (-7.17 to 1.73) | 0.231 |
| *3* | -15.55 (-21.68 to -9.42) | <0.001 |
| *4* | -14.98 (-19.18 to -10.78) | <0.001 |
| *5* | -13.49 (-17.00 to -9.98) | <0.001 |
| **ATTEMPT NUMBER** |  |  |
| *First attempt of UCAT* | 0 |  |
| *Second or more than second attempt of UCAT* | 3.18 (-0.01 to 6.36) | 0.051 |
| **BURSARY STATUS** |  |  |
| *No bursary received* | 0 |  |
| *Bursary* | -3.64 (-6.69 to -0.58) | 0.020 |
| *Constant* | 662.21 (660.41 to 664.02) | <0.001 |

**Quantitative Reasoning**

| **Variable** | **Mean difference (95% CI)** | **p-value** |
| --- | --- | --- |
| **GENDER** |  |  |
| *Female* | 0 |  |
| *Male* | 29.53 (26.51 to 32.55) | <0.001 |
| **SCHOOL TYPE** |  |  |
| *Sixth Form/Further Education College* | 0 |  |
| *Grammar* | 37.92 (34.00 to 41.83) | <0.001 |
| *Independent/ Private Fee Paying* | 28.34 (24.25 to 32.44) | <0.001 |
| *Comprehensive* | 10.86 (6.04 to 15.68) | <0.001 |
| *Other* | -9.67 (-23.35 to 4.00) | 0.166 |
| **ETHNICITY** |  |  |
| *White* | 0 |  |
| *Asian* | -16.10 (-19.47 to -12.72) | <0.001 |
| *Black* | -62.38 (-68.12 to -56.64) | <0.001 |
| *Mixed* | -15.26 (-22.00 to -8.52) | <0.001 |
| *Other* | -27.27 (-35.17 to -19.38) | <0.001 |
| **NS-SEC** |  |  |
| *1* | 0 |  |
| *2* | -4.26 (-11.86 to 3.35) | 0.273 |
| *3* | -21.66 (-32.14 to -11.19) | <0.001 |
| *4* | -24.27 (-31.44 to -17.09) | <0.001 |
| *5* | -20.00 (-25.99 to -14.00) | <0.001 |
| **ATTEMPT NUMBER** |  |  |
| *First attempt of UCAT* | 0 |  |
| *Second or more than second attempt of UCAT* | 18.00 (12.56 to 23.44) | <0.001 |
| **BURSARY STATUS** |  |  |
| *No bursary received* | 0 |  |
| *Bursary* | -5.02 (-10.23 to 0.20) | 0.059 |
| *Constant* | 699.69 (696.60 to 702.77) | <0.001 |

**Verbal Reasoning**

| **Variable** | **Mean difference (95% CI)** | **p-value** |
| --- | --- | --- |
| **GENDER** |  |  |
| *Female* | 0 |  |
| *Male* | 11.54 (8.97 to 14.11) | <0.001 |
| **SCHOOL TYPE** |  |  |
| *Sixth Form/Further Education College* | 0 |  |
| *Grammar* | 25.20 (21.87 to 28.53) | <0.001 |
| *Independent/ Private Fee Paying* | 23.63 (20.15 to 27.11) | <0.001 |
| *Comprehensive* | 6.83 (2.73 to 10.93) | 0.001 |
| *Other* | -5.18 (-16.81 to 6.45) | 0.382 |
| **ETHNICITY** |  |  |
| *White* | 0 |  |
| *Asian* | -32.36 (-35.23 to -29.49) | <0.001 |
| *Black* | -40.58 (-45.46 to -35.70) | <0.001 |
| *Mixed* | -11.60 (-17.33 to -5.87) | <0.001 |
| *Other* | -38.21 (-44.92 to -31.50) | <0.001 |
| **NS-SEC** |  |  |
| *1* | 0 |  |
| *2* | -0.70 (-7.17 to 5.76) | 0.831 |
| *3* | -11.44 (-20.35 to -2.53) | 0.012 |
| *4* | -17.33 (-23.43 to -11.23) | <0.001 |
| *5* | -19.20 (-24.30 to -14.10) | <0.001 |
| **ATTEMPT NUMBER** |  |  |
| *First attempt of UCAT* | 0 |  |
| *Second or more than second attempt of UCAT* | 6.51 (1.88 to 11.14) | 0.006 |
| **BURSARY STATUS** |  |  |
| *No bursary received* | 0 |  |
| *Bursary* | -3.67 (-8.11 to 0.76) | 0.104 |
| *Constant* | 582.05 (579.43 to 584.67) | <0.001 |

## Appendix 4: SJT Band 4 binary logistic regression models with demographic variables as covariates

A binary logistic model was fitted with the lowest Band (Band 4) in the SJT as the outcome (compared to the other three Bands) and the results are similar to those of Table 12, demonstrating that candidates who are male, of ‘non-White’ ethnicity, attending sixth forms or colleges or are sitting the test for the first time were significantly more likely to score a Band 4. With regards to socioeconomic status, students from more deprived families (those with an NS-SEC 3 to 5) were significantly more likely to score a Band 4 than those who are least deprived (NS-SEC of 1).

*Binary logistic regression model with SJT Band 4 as the outcome using all eligible candidates. Adjusted odds ratios, relative to a reference category for each variable and adjusted for all other included variables, are presented here for each category.*

| **Variable** | **Adjusted odds ratio (95% CI)** | **p-value** |
| --- | --- | --- |
| **GENDER** |  |  |
| *Female* | 1 |  |
| *Male* | 1.91 (1.64 to 2.23) | <0.001 |
| **SCHOOL TYPE** |  |  |
| *Sixth Form/Further Education College* | 1 |  |
| *Grammar* | 0.51 (0.41 to 0.65) | <0.001 |
| *Independent/ Private Fee Paying* | 0.64 (0.50 to 0.81) | <0.001 |
| *Comprehensive* | 0.82 (0.63 to 1.08) | 0.160 |
| *Other* | 0.72 (0.31 to 1.66) | 0.443 |
| **ETHNICITY** |  |  |
| *White* | 1 |  |
| *Asian* | 2.92 (2.40 to 3.54) | <0.001 |
| *Black* | 3.33 (2.55 to 4.37) | <0.001 |
| *Mixed* | 1.64 (1.10 to 2.44) | 0.015 |
| *Other* | 2.97 (2.07 to 4.25) | <0.001 |
| **NS-SEC** |  |  |
| *1* | 1 |  |
| *2* | 0.67 (0.41 to 1.11) | 0.117 |
| *3* | 1.63 (1.05 to 2.51) | 0.028 |
| *4* | 1.53 (1.14 to 2.06) | 0.005 |
| *5* | 1.47 (1.15 to 1.88) | 0.002 |
| **ATTEMPT NUMBER** |  |  |
| *First attempt of UCAT* | 1 |  |
| *Second or more than second attempt of UCAT* | 0.28 (0.18 to 0.45) | <0.001 |
| **BURSARY** |  |  |
| *No bursary received* | 1 |  |
| *Bursary received* | 0.93 (0.73 to 1.19) | 0.577 |
| *Constant* | 0.03 (0.02 to 0.03) | <0.001 |

## Appendix 5: Subsection score regression models with demographic variables and preparedness categories as covariates

Multiple linear regression models are presented below with each subsection score individually as the outcome and preparedness categories and demographic variables as covariates. The coefficient for each included variable in the model is equivalent to the mean difference in the corresponding subsection score for that category compared to a reference category when adjusting for the other included covariates. The reference category for each variable has a coefficient of zero. For each of the preparedness categories, the reference category is not being in the category. For example, for ‘paid preparation materials’, the reference category is those who did not report using paid preparation materials.

**Abstract Reasoning**

| Variable | Mean difference (95% CI) | p-value |
| --- | --- | --- |
| GENDER |  |  |
| *Female* | 0 |  |
| *Male* | 7.22 (2.60 to 11.84) | 0.002 |
| **SCHOOL TYPE** |  |  |
| *Sixth Form/Further Education College* | 0 |  |
| *Grammar* | 28.13 (22.01 to 34.25) | <0.001 |
| *Independent/ Private Fee Paying* | 27.75 (21.40 to 34.09) | <0.001 |
| *Comprehensive* | 3.45 (-3.53 to 10.44) | 0.332 |
| *Other* | -4.83 (-23.13 to 13.46) | 0.605 |
| **ETHNICITY** |  |  |
| *White* | 0 |  |
| *Asian* | -15.01 (-20.26 to -9.76) | <0.001 |
| *Black* | -45.88 (-54.02 to -37.73) | <0.001 |
| *Mixed* | -7.99 (-17.97 to 1.99) | 0.116 |
| *Other* | -15.01 (-27.70 to -2.31) | 0.021 |
| **NS-SEC** |  |  |
| *1* | 0 |  |
| *2* | -13.09 (-24.17 to -2.01) | 0.021 |
| *3* | -11.89 (-29.77 to 5.99) | 0.193 |
| *4* | -13.35 (-24.44 to -2.25) | 0.018 |
| *5* | -17.09 (-26.07 to -8.12) | <0.001 |
| **Second or more than second attempt of UCAT** | 24.46 (15.62 to 33.31) | <0.001 |
| **School-based preparation course** | 5.49 (-0.57 to 11.55) | 0.076 |
| **Official UCAT Tests** | 15.99 (10.17 to 21.82) | <0.001 |
| **Other official UCAT resources** | -2.47 (-7.23 to 2.28) | 0.308 |
| **Free commercial materials** | 0.88 (-5.75 to 7.50) | 0.795 |
| **Paid commercial materials** | 19.32 (14.57 to 24.06) | <0.001 |
| **TIME PREPARING** |  |  |
| *Did not prepare* | 0 |  |
| *0-10 hours* | 21.33 (-7.14 to 49.80) | 0.142 |
| *11-20 hours* | 45.31 (17.37 to 73.26) | 0.001 |
| *21-30 hours* | 52.67 (24.76 to 80.57) | <0.001 |
| *31-40 hours* | 65.45 (37.44 to 93.45) | <0.001 |
| *40+ hours* | 74.05 (46.06 to 102.04) | <0.001 |
| **BURSARY STATUS** |  |  |
| *No bursary received* | 0 |  |
| *Bursary received* | -2.23 (-10.13 to 5.67) | 0.58 |
| *Constant* | 558.36 (529.93 to 586.79) | <0.001 |

**Decision Making**

| Variable | Mean difference (95% CI) | p-value |
| --- | --- | --- |
| **GENDER** |  |  |
| *Female* | 0 |  |
| *Male* | 5.74 (2.83 to 8.65) | <0.001 |
| **SCHOOL TYPE** |  |  |
| *Sixth Form/Further Education College* | 0 |  |
| *Grammar* | 17.06 (13.20 to 20.92) | <0.001 |
| *Independent/ Private Fee Paying* | 18.48 (14.48 to 22.48) | <0.001 |
| *Comprehensive* | 1.77 (-2.63 to 6.18) | 0.43 |
| *Other* | -13.66 (-25.21 to -2.12) | 0.02 |
| **ETHNICITY** |  |  |
| *White* | 0 |  |
| *Asian* | -25.16 (-28.47 to -21.85) | <0.001 |
| *Black* | -41.76 (-46.90 to -36.62) | <0.001 |
| *Mixed* | -14.07 (-20.36 to -7.78) | <0.001 |
| *Other* | -29.91 (-37.92 to -21.90) | <0.001 |
| **NS-SEC** |  |  |
| *1* | 0 |  |
| *2* | -6.97 (-13.96 to 0.01) | 0.050 |
| *3* | -8.71 (-19.99 to 2.57) | 0.130 |
| *4* | -10.46 (-17.46 to -3.47) | 0.003 |
| *5* | -12.69 (-18.35 to -7.03) | <0.001 |
| **Second or more than second attempt of UCAT** | 7.26 (1.68 to 12.84) | 0.011 |
| **School-based preparation course** | 8.03 (4.21 to 11.86) | <0.001 |
| **Official UCAT Tests** | 10.67 (6.99 to 14.34) | <0.001 |
| **Other official UCAT resources** | 0.86 (-2.14 to 3.86) | 0.576 |
| **Free commercial materials** | -4.37 (-8.55 to -0.19) | 0.040 |
| **Paid commercial materials** | 0.32 (-2.67 to 3.31) | 0.834 |
| **TIME PREPARING** |  |  |
| *Did not prepare* | 0 |  |
| *0-10 hours* | 10.98 (-6.98 to 28.94) | 0.231 |
| *11-20 hours* | 19.40 (1.77 to 37.02) | 0.031 |
| *21-30 hours* | 18.22 (0.62 to 35.82) | 0.042 |
| *31-40 hours* | 20.74 (3.08 to 38.41) | 0.021 |
| *40+ hours* | 20.20 (2.54 to 37.86) | 0.025 |
| **BURSARY STATUS** |  |  |
| *No bursary received* | 0 |  |
| *Bursary received* | -3.92 (-8.90 to 1.07) | 0.123 |
| *Constant* | 639.99 (622.06 to 657.92) | <0.001 |

**Quantitative Reasoning**

| Variable | Mean difference (95% CI) | p-value |
| --- | --- | --- |
| **GENDER** |  |  |
| *Female* | 0 |  |
| *Male* | 29.76 (24.84 to 34.68) | <0.001 |
| **SCHOOL TYPE** |  |  |
| *Sixth Form/Further Education College* | 0 |  |
| *Grammar* | 37.41 (30.89 to 43.92) | <0.001 |
| *Independent/ Private Fee Paying* | 21.61 (14.85 to 28.37) | <0.001 |
| *Comprehensive* | 7.14 (-0.30 to 14.58) | 0.060 |
| *Other* | -13.36 (-32.85 to 6.13) | 0.179 |
| **ETHNICITY** |  |  |
| *White* | 0 |  |
| *Asian* | -27.22 (-32.81 to -21.63) | <0.001 |
| *Black* | -70.83 (-79.51 to -62.16) | <0.001 |
| *Mixed* | -15.76 (-26.39 to -5.13) | 0.004 |
| *Other* | -38.03 (-51.55 to -24.52) | <0.001 |
| **NS-SEC** |  |  |
| *1* | 0 |  |
| *2* | 1.61 (-10.19 to 13.41) | 0.789 |
| *3* | -21.93 (-40.97 to -2.88) | 0.024 |
| *4* | -16.87 (-28.69 to -5.06) | 0.005 |
| *5* | -12.96 (-22.53 to -3.40) | 0.008 |
| **Second or more than second attempt of UCAT** | 21.57 (12.15 to 30.99) | <0.001 |
| **School-based preparation course** | 11.66 (5.20 to 18.11) | <0.001 |
| **Official UCAT Tests** | 21.73 (15.53 to 27.93) | <0.001 |
| **Other official UCAT resources** | -1.36 (-6.42 to 3.71) | 0.599 |
| **Free commercial materials** | -2.57 (-9.63 to 4.48) | 0.475 |
| **Paid commercial materials** | 13.02 (7.97 to 18.07) | <0.001 |
| **TIME PREPARING** |  |  |
| *Did not prepare* | 0 |  |
| *0-10 hours* | 17.29 (-13.03 to 47.61) | 0.264 |
| *11-20 hours* | 35.03 (5.27 to 64.79) | 0.021 |
| *21-30 hours* | 36.34 (6.62 to 66.06) | 0.017 |
| *31-40 hours* | 51.45 (21.62 to 81.28) | 0.001 |
| *40+ hours* | 59.91 (30.10 to 89.72) | <0.001 |
| **BURSARY STATUS** |  |  |
| *No bursary received* | 0 |  |
| *Bursary received* | -3.66 (-12.07 to 4.76) | 0.394 |
| *Constant* | 641.53 (611.25 to 671.81) | <0.001 |

**Verbal Reasoning**

| Variable | Mean difference (95% CI) | p-value |
| --- | --- | --- |
| **GENDER** |  |  |
| *Female* | 0 |  |
| *Male* | 10.05 (5.75 to 14.35) | <0.001 |
| **SCHOOL TYPE** |  |  |
| *Sixth Form/Further Education College* | 0 |  |
| *Grammar* | 29.97 (24.27 to 35.67) | <0.001 |
| *Independent/ Private Fee Paying* | 26.37 (20.46 to 32.28) | <0.001 |
| *Comprehensive* | 3.81 (-2.70 to 10.31) | 0.251 |
| *Other* | -15.24 (-32.28 to 1.80) | 0.080 |
| **ETHNICITY** |  |  |
| *White* | 0 |  |
| *Asian* | -36.48 (-41.36 to -31.59) | <0.001 |
| *Black* | -46.82 (-54.40 to -39.23) | <0.001 |
| *Mixed* | -17.88 (-27.18 to -8.59) | <0.001 |
| *Other* | -46.39 (-58.21 to -34.57) | <0.001 |
| **NS-SEC** |  |  |
| *1* | 0 |  |
| *2* | -3.94 (-14.26 to 6.37) | 0.454 |
| *3* | -8.46 (-25.11 to 8.19) | 0.319 |
| *4* | -9.86 (20.19 to -0.47) | 0.061 |
| *5* | -21.77 (-30.13 to -13.41) | <0.001 |
| **Second or more than second attempt of UCAT** | 13.35 (5.11 to 21.59) | 0.001 |
| **School-based preparation course** | 11.48 (5.83 to 17.12) | <0.001 |
| **Official UCAT Tests** | 19.38 (13.96 to 24.80) | <0.001 |
| **Other official UCAT resources** | 1.74 (-2.69 to 6.17) | 0.441 |
| **Free commercial materials** | -7.88 (-14.05 to -1.71) | 0.012 |
| **Paid commercial materials** | 5.00 (0.58 to 9.41) | 0.027 |
| **TIME PREPARING** |  |  |
| *Did not prepare* | 0 |  |
| *0-10 hours* | 18.87 (-7.64 to 45.38) | 0.163 |
| *11-20 hours* | 24.28 (-1.74 to 50.29) | 0.067 |
| *21-30 hours* | 24.53 (-1.45 to 50.51) | 0.064 |
| *31-40 hours* | 28.24 (2.16 to 54.32) | 0.034 |
| *40+ hours* | 23.74 (-2.32 to 49.81) | 0.074 |
| **BURSARY STATUS** |  |  |
| *No bursary received* | 0 |  |
| *Bursary received* | -2.32 (-9.67 to 5.04) | 0.537 |
| *Constant* | 550.83 (524.36 to 577.30) | <0.001 |
